# Challenges and pathways to herbal ethical approval: an institutional case study of a randomized controlled trial on *Moringa oleifera* Lam.

**DOI:** 10.3389/fphar.2026.1881552

**Published:** 2026-06-24

**Authors:** Aisha Gambo, Nceba Gqaleni, Limakatso Lebina

**Affiliations:** 1 Sub-Discipline of Traditional Medicine, School of Medicine, College of Health Sciences, University of KwaZulu-Natal, Durban, South Africa; 2 Africa Health Research Institute, KwaZulu-Natal, South Africa

**Keywords:** clinical trials, ethics review, herbal medicines, Moringa oleifera, Nigeria, regulatory framework, South Africa

## Abstract

Clinical trials are essential for validating the safety and efficacy of traditional medicines; however, their conduct in Africa is significantly hindered by resource constraints, a lack of standardized regulatory frameworks, and insufficient guidance for navigating complex ethical review processes. This study addresses the knowledge gap regarding the practical experience of obtaining multi-country regulatory and ethical approvals for herbal clinical trials. A qualitative case study design was employed to document the regulatory and ethical review processes for a randomized controlled trial of *Moringa oleifera* Lam. leaf supplementation in HIV-positive adults receiving antiretroviral therapy. Data were synthesized from institutional correspondence, ethical reviews, and communication records with the University of KwaZulu-Natal in South Africa, Aminu Kano Teaching Hospital, and the National Agency for Food and Drug Administration and Control in Nigeria. This study highlights the challenges of managing multi-country regulatory systems, particularly in the absence of harmonized guidelines for herbal research. The findings suggest that inter-institutional collaboration and adherence to rigorous Good Clinical Practice (GCP) standards are critical for the successful conduct of herbal trials. The review process revealed significant differences in turnaround times, documentation requirements, and query complexities across the involved institutions. In conclusion, this study emphasizes the need for regionally harmonized, risk-based regulatory frameworks for herbal medicines. Sharing practical experience is vital for building capacity. This enables researchers to better navigate institutional bureaucracies and advance high-quality clinical research in developing countries.

## Introduction

Clinical trials (CT) play a crucial role in the development of standardized phytomedicines that are safe, efficacious, and of optimum quality (Kasilo et al., 2019). Clinical Trials are defined as studies of new tests and treatments that evaluate their effects on human health outcomes, conducted according to approved guidelines (WHO, 2022).

In Africa, various challenges impede the conduct of clinical trials of herbal medicines. This includes a lack of clinical data on the safety, efficiency, and quality of traditional medicines, alongside resource constraints (both financial and human) (Mutombo et al., 2023). Given the peculiarities of herbal medicines, there is inevitably an inadequate regulatory framework for the review of herbal protocols and the conduct of herbal trials across various African countries (Sangho et al., 2024). Thus, clinical investigators and ethics committee members often lack the confidence required to address related ethical issues (Koonrungsesomboon et al., 2021).

To assist researchers in conducting ethical herbal trials in developing countries, the literature has reported a greater focus on enhancing the research capacity of researchers and mobilizing research funds (Monera-Penduka et al., 2017). Limited attention has been given to sharing the experiences involved in the successful ethical and regulatory review processes for clinical trials of herbal medicines (Monera-Penduka et al., 2017). To improve health outcomes, clinical research on herbal medicines should be designed to provide innovative, beneficial, and generalizable knowledge while protecting study participants (Monera-Penduka et al., 2017). A balance between scientific validity and ethics must be achieved. The literature is lacking on the experiences and procedures regarding the ethical and regulatory processes of researchers involved in clinical trials of herbal medicines in developing countries. Dissemination of such information to the public will offer a platform for other researchers to learn and build their capacity.

A randomized controlled trial was conducted in 2017 to evaluate the impact of *Moringa oleifera* Lam. leaf supplementation on anthropometric parameters and immune status in adult PLHIV on ART at Aminu Kano Teaching Hospital (AKTH), Kano State, Nigeria. Two hundred HIV-positive patients were randomly allocated to either the *Moringa Oleifera* group (MOG) or the control group (COG). Changes in anthropometric parameters (weight and body mass index [BMI]) and CD4 cell counts were measured monthly for 6 months, while HIV-1 viral loads were measured at baseline and the end of the study for both groups. The clinical trial was registered with the Pan-African Clinical Trial Registry under the identification number PACTR201811722056449 (Gambo et al., 2021).

Due to limited knowledge, researchers have found it challenging to obtain useful information on the successful regulatory review process required for conducting clinical trials of herbal medicines. Consequently, the authors realized the need to report this aspect of the study. Therefore, this study aims to share our experience with the ethical and regulatory review processes involved and the timeline required to obtain the necessary approvals. Information and dialogues with institutional and regulatory authorities are summarized and discussed.

This study adopted a case study design. A qualitative case study provides an in-depth understanding of the developmental processes within a specific context. A case study can be beneficial in offering a voice to underrepresented experiences. It can be used to transmit knowledge within communities of practice. As such, the case study design methodology contributes to the development of professional expertise and clinical flexibility by integrating theoretical knowledge with practical application (Hwong et al., 2022; Råbu and Binder, 2025). Data was collected using documents involved in the ethical review process. The data used is highly authentic and offers primary source evidence. A categorical analytical framework was used to organize, process, and evaluate the information obtained from correspondence documents, institutional communications, and review records by grouping them into distinct categories to ensure precise, systematic mapping of institutional regulatory milestones (Bowen, 2009). The data was further analyzed and synthesized to understand the dataset (Lochmiller, 2021). This approach provides an in-depth understanding of the regulatory decision-making process in a real-life context (Paparini et al., 2020). Therefore, the following three categories were formed as a basis for evaluating the ethical and regulatory processes: (1) Regulatory documentation and compliance; (2) Review and query resolution procedures; and (3) Institutional procedure and comparison.

## Ethical and regulatory processes involved in the study

The clinical trial was funded by the Department of Science and Innovation of South Africa and the University of KwaZulu-Natal (UKZN), which is the institution of the Principal Investigator (PI). Owing to the lack of a specific framework and guidelines for conducting clinical trials of herbal medicines in South Africa, this study was conducted in Nigeria. Therefore, this study required ethical and regulatory approvals from two countries: Nigeria and South Africa. The ethical rationale for dual country oversight was considered. This is to ensure that trials funded by developed countries do not take advantage of weak regulatory standards in low-income host countries. Beneficence in research ethics seeks to maximize research benefits for society and participants while minimizing the risks (McNair, 2022). Hence, South African regulatory processes were heavily involved to ensure that the rights and welfare of the participants were protected (McNair, 2022).

The Biomedical Research Ethics Committee (BREC) of the University of KwaZulu-Natal, Durban, South Africa, provided institutional approval for this study. Approval was granted under reference number BFC294/16. Approval was not required from the South African Health Products Regulatory Authority (SAHPRA) (formerly part of the Medicines Control Council (MCC) because the clinical trial was not conducted in South Africa. Initially, an application was submitted to the Ethics Committee of AKTH, Nigeria, seeking permission to conduct the study at the S. S. Wali Virology Centre. Permission was granted pending institutional ethics approval from the BREC at UKZN, South Africa, and further ethics approval from the ethics committee of AKTH.

In Nigeria, institutional approval was obtained from the Ethics Committee of AKTH, Kano, Nigeria (reference number: NHREC/21/08/2008/AKTH/EC/2012). Further approval was sought from the National Agency for Food and Drug Administration and Control (NAFDAC), the regulatory authority responsible for approving all clinical trials conducted in Nigeria, including those of herbal medicines. NAFDAC aims to supervise the manufacture, importation, distribution, sale, and use of herbal products in Nigeria by developing guidelines and regulatory documents to ensure that they meet safety, efficacy, and quality standards (NAFDAC, 2020). The agency draws its mandate under the Herbal Medicines and Related Products (Registration) Regulations, 2021, by sections 5 and 30 of the National Agency for Food and Drug Administration and Control Act (Cap NI LFN) 2004, and Section 12 of the Food, Drug, and Related Products (Registration, etc*.*) Act Cap F33 LFN 2004 (NAFDAC, 2021).

## Procedures for review of the herbal protocol

At the onset, correspondence with NAFDAC established that the procedure for the approval of clinical trials of herbal medicines is the same as that for conventional medicines because of the lack of a specific framework for herbal clinical trials. We followed the recommended framework for the conventional trials. Before an application to conduct a conventional trial can be submitted to the NAFDAC, the protocol must undergo an ethical review by the ethics committees of both institutions responsible for the trial.

Therefore, as recommended, the herbal protocol was first submitted to the Biomedical Research Ethics Committee (BREC) of the University of KwaZulu-Natal (UKZN) in South Africa. The BREC is registered with the South African Department of Health’s National Health Research Ethics Council (http://nhrec.health.gov.za). It is also registered with the US Office for Human Research Protections (http://www.hhs.gov/ohrp/) (OHRP) and has a Federal-Wide Assurance (FWA). All health-related biomedical and social research at the University of KwaZulu-Natal requires prior ethical clearance from the BREC (BREC, 2026). The BREC either approves research proposals as submitted, approves them subject to specified conditions, or rejects proposals based on the current UKZN Research Ethics Policy, BREC Terms of Reference, Standard Operating Procedures, and applicable guidance (BREC, 2026).

The herbal protocol for our study was submitted to the BREC for review and ethical approval.

Upon approval, an application to conduct the study at AKTH, Nigeria, was submitted along with the herbal protocol for review by the Research Ethics Committee of AKTH. Ethics approval was granted 3 weeks after the application. The approval was subject to periodic reporting of the study’s progress and completion to the REC. Co-supervision of an expert in HIV and AKTH staff was sought. The co-supervisor supervised and monitored the clinical trials at the study site.

The study protocol was submitted simultaneously to the NAFDAC and REC of AKTH. Nevertheless, approval was obtained from the AKTH before the NAFDAC issued the final authorization for the conduct of the trial. Ethically, all clinical trial protocols must be reviewed and approved by NAFDAC, including academic clinical trials for herbal formulations for which the safety/efficacy profile has not been determined, especially in the Nigerian population (NAFDAC, 2024). The NAFDAC is responsible for protocol review and authorization of clinical trials before they are conducted in Nigeria. It is also responsible for carrying out inspections of trial sites to monitor the conduct of authorized studies to ensure that the wellbeing and safety of the participants are protected and that credible data are obtained from the study (NAFDAC, 2024).

Other procedures involved in the review and approval require the payment of a clinical trial approval fee to NAFDAC. After a satisfactory review of the protocol, approval was granted to conduct a clinical trial of *M. oleifera* at the S. S. Wali Virology Centre, AKTH. During the study, a Good Clinical Practice (GCP) inspection of the trial was conducted by NAFDAC GCP Inspectors. The inspection was conducted in accordance with the NAFDAC Act Cap N1 LFN, 2004, and the Food, Drugs, and Related Products (Registration, etc*.*) Act Cap F33, Section 5 (1) and 5 (2). The inspection plan included a document review and visits to the facilities involved in the clinical study. This includes pharmacy and clinical laboratories. The inspectors requested that all relevant documentation, including study-related files, procedures, Case Report Forms, Source documents, and medical records, be made available and accessible to enable the inspectors to have direct access to the records.

Finally, on completion of the clinical trial, the REC of AKTH and NAFDAC were notified of the results.

## Preparation of documents to support the herbal protocol application

To support protocol application, relevant documents, analyses, and procedures were conducted, obtained, and/or established. This includes reports confirming the taxonomic identity of *M. oleifera* Lam. Leaf obtained from the Herbarium of the Department of Biological Sciences, Bayero University Kano (BUK), Nigeria. Second, a Repeated Dose 90-day oral toxicity study of *M. oleifera* Lam. dried leaf powder to determine the acute oral toxicity of the powder. No adverse effects were observed. The study was conducted by La-Bio Research (South Africa).

Furthermore, samples of *M. oleifera* Lam. leaf powder (Nigerian ecotype) and the study placebo were transported to analyze their nutritional content. This was conducted by the South African National Accreditation System (SANAS)-accredited ASPIRATA Food and Beverage Laboratory. A professional Indemnity with Niger Insurance, Kano State, Nigeria, was also obtained. Lastly, the study was registered with the Pan-African Clinical Trial Registry under the identification number PACTR201811722056449.

The above documents either supported the initial protocol application or were requested during the protocol review.

**FIGURE 1:**
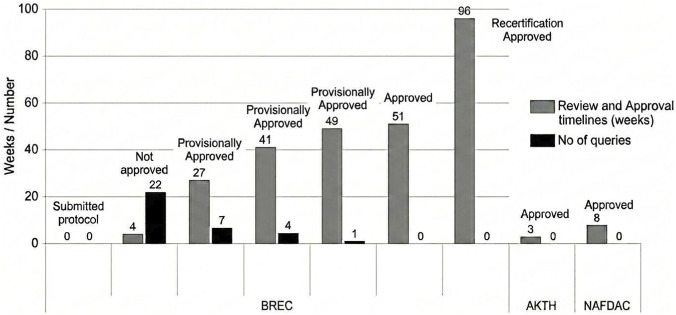
Duration, number of queries and status of protocol review across institutions.

Table 1 shows a comparison of the duration, number of queries, and documentation requirements and Figure 1 shows a snapshot of the duration, number of queries, and status of protocol review across the various institutions. There was a significant variation in the duration and number of queries across institutions. The BREC (UKZN) was the first ethics committee to review the protocol. It had the longest review duration with the highest number of queries (96 weeks; 33 queries). As such, many documents were requested during the review process, some of which took a while to obtain. An example is the 90-day oral toxicity study; reports of nutritional content analysis and the evidence of PACTR registration. An extensive ethical review is conducted to avoid exploitation, protect vulnerable populations, and ensure research integrity (Wendler, 2017). Moreover, there is strict adherence to rigid administrative requirements, which often lengthens the review processes (Aung et al., 2020). After a satisfactory review of the submitted documents, the REC of AKTH also used international standards while adapting the review to the local cultural context and granted approval within 3 weeks with no queries (Zumla and Costello, 2002). There is less rigidity to minor administrative errors or inconsistencies in the review of documents, which assisted in hastening the review processes and approval (Aung et al., 2020).

**TABLE 1:** Comparison of duration, number of queries, and documentation requirements across institutions.

Institution	Duration (weeks)	No of queries	Documentation requirements
BREC	96	33	- CV and ethics training certificate of supervisor
			- Reports of the taxonomic identity of the *Moringa oleifera* lam. leaf
			- Repeated dose 90-day oral toxicity study of the *Moringa oleifera* lam. dried leaf powder
			- Reports of nutritional content analysis of *Moringa oleifera* lam. leaf powder (Nigerian ecotype) and the study placebo
			- Evidence of PACTR registration
AKTH	3	0	- All documents submitted to BREC
			- Letter of permission to conduct the study at the virology centre of AKTH
			- Approval letter from BREC
			- Approval letter of Nigerian co-supervisor by BREC
			- Evidence of professional Indemnity with Niger Insurance, kano state, Nigeria
NAFDAC	8	0	- All documents submitted to AKTH
			- Evidence of payment of NAFDAC clinical trial approval fee

## Feedback on selected queries from BREC and responses made

During the Herbal protocol review with BREC, several queries were raised, and appropriate responses were offered. In the first response from the BREC, ethics training certificates for the supervisor were requested. Although our study was an herbal trial, GCP and ethics certificates were mandatory for all research members. The principles of GCP address the design, ethics, and reporting of clinical trials. This ensures that the rights and safety of human participants are protected. It also ensures that clinical trials are conducted according to approved protocols with integrity and rigor (NAFDAC, 2024; NIH, 2024).

Two further queries were stated: *This is a clinical trial and therefore will need to be registered with the South African National Clinical Trials Register (SANCTR)*’’, and ‘‘*The Medicines Control Council (MCC) approval is required based on MCC guidance that “Complementary or alternative medicines making claims for and purporting to treat, diagnose, and modify conditions of HIV and AIDS, diabetes, hypertension and cancer should submit an application for evaluation to the council in accordance with the requirements of the General Guidance on Complementary Medicine Registration*”. The absence of a specific framework to guide researchers and regulators in conducting clinical trials of herbal products made it challenging to address these queries. At the beginning of the review process, our understanding of which regulatory bodies we needed to apply for approval was minimal. Therefore, in response to the two queries, we reported that “*The study will be registered with SANCTR after we receive institutional approval from UKZN*’’ and “*Application for evaluation of the study will be submitted to MCC, and approval will be obtained after we receive institutional approval from UKZN*”.

Other queries related to the investigational product included the following: “*Known allergy to the product under study/investigation is exclusionary for study participation. Based on the literature reviewed, can the PI please describe the type and severity of the reaction that may occur? This information should be provided in the participant information leaflet/informed consent document as a potential risk factor*”. In addition, “*The investigational product has the potential to interact with ARVs via the CYP 3A4 system. Please advise on the schedule for the evaluation of viral load/CD4 during study participation. Is there any committee that will monitor safety in the form of a PSRT/CMC/DSMB?*’‘. Our response was “*Any adverse events will be closely monitored. In Nigeria, all tertiary health institutions that operate daily HIV clinics have been equipped to monitor adverse drug reactions (ADRs). The Global HIV/AIDS Initiative Nigeria (GHAIN) and NAFDAC developed a structured ADR screening form modified from the WHO (* Eluwa et al., 2012 *). Clinicians and pharmacists are trained in the content and use of the form. They are required to use the form for all patients on* ARV *at every clinical visit. Therefore, in our study, we propose adopting this form to capture adverse events. The DSMB committee will monitor and ensure the safety of the study participants and data”*.

In the second response from BREC, a query stated that “*MCC approval needs to be applied for in parallel with BREC application; need not await BREC approval*”. At the time this query was received, both the results of the 90-day acute toxicity study of *Moringa oleifera* Lam. dried leaf powder and the analysis of the nutritional content of *M. oleifera* had not concluded. Therefore, we responded as such and added “*On getting the results, we will submit our application to conduct a clinical trial and all other documents required to the MCC and will provide feedback to BREC when MCC approval is granted*”. However, before the next correspondence from the BREC, we received the correct information that MCC permission and SANCTR registration would not be needed for our study, as it would not be conducted in South Africa. This information was conveyed to BREC in response to a further query, which stated that “*MCC permission pending*”. We also added that “*As this study is going to be conducted in Nigeria, permission and approval to conduct the study will be obtained from the Aminu Kano Teaching Hospital, Kano, Nigeria, and authorization will be sought from NAFDAC*”. This study was registered with the PACTR.

Thereafter, the BREC approval was granted, subject to the condition that approval from Nigeria be forwarded to BREC as soon as possible. Additionally, the approval letter lists the committee members who approved the study. It was a 14-member meeting that included experts from different disciplines, such as neurosurgery, surgery, obstetrics and gynecology, HIV medicine, and external community members.

To compare the regulatory framework in other African countries, a study conducted in Zimbabwe reported the shortest review turnaround time by the National Ethics Committee (NEC). This was attributed to an improved review efficiency by the NEC, as it is the only committee that has published turnaround times for the review of applications in Zimbabwe (Monera-Penduka et al., 2017). This is similar to our reported protocol review experience with AKTH, where the review process was the shortest. The UKZN BREC approval, together with the rigorous regulatory oversight of the AKTH and the National Health Research Ethics Committee (NHREC), facilitated the review process. The NHREC is largely responsible for regulating other institutional ethics committees in Nigeria (Yakubu and Adebamowo, 2013). Contrary to our review experience with the NAFDAC, their deliberations with the Medicines Control Authority of Zimbabwe (MCAZ), which is the National Drug Regulatory Authority (NDRA), took over a year. The NDRA approved and monitored all clinical trials in Zimbabwe. It is responsible for the thorough assessment of the existing evidence and quality control (Monera-Penduka et al., 2017). In our study, the NAFDAC review took 8 weeks, and approval was granted only after examining all submitted documents and institutional approval from AKTH was obtained. The extended intensive review by the BREC of UKZN reflects the ethical rationale for multi-country trials. This is to ensure that cross-border ethics are adhered to and to avoid any unfavorable institutional bureaucracy between the funding and the host institution (Canario Guzmán et al., 2017).

## Post-study regulatory developments, recommendations, and limitations

Several developments have occurred in various African countries since the conduct of our clinical trial. Recently, NAFDAC partnered with the Nigeria Natural Medicine Development Agency (NNMDA) to design clinical trials for the development of scientifically proven, safe, and effective herbal medicines that meet international standards (PUNCH, 2025). In South Africa, the SAHPRA was established as an independent entity in February 2018, replacing the Medicines Control Council (MCC) and Directorate of Radiation Control (DRC) (SAHPRA, 2025).

The role of ethics committees, regulatory agencies, and policymakers in Africa in standardizing herbal trial review pathways is to encourage the research and development of innovative herbal products. We recommend that this be achieved by establishing a harmonized joint review platform with regionally harmonized frameworks. This will prevent the review of herbal protocols in isolation, which leads to systemic delays. In addition, a risk-based approach for herbal medicine review should be adopted, where products are categorized based on their level of scientific development. This will allow regulators to apply rigorous ethical oversight to high-risk products while maintaining simplified pathways for products with long histories of traditional use (Gambo et al., 2025).

This study reports an individual institutional experience; therefore, as a limitation, the findings of our study are context-specific and may not fully represent regulatory experiences across different African countries or institutions.

## Conclusion

There is a knowledge void, as researchers find it challenging to obtain useful information on the successful regulatory review process necessary for conducting clinical trials of herbal medicines. Dissemination of such information to the public alongside the main clinical study findings will offer a platform for other researchers to learn and build their capacity.

## Data Availability

The raw data supporting the conclusions of this article will be made available by the authors, without undue reservation.
